# Let’s talk about “talking” dogs! Reviewing the science behind a bold idea

**DOI:** 10.1007/s42977-025-00276-0

**Published:** 2025-07-29

**Authors:** Rita Lenkei, Paula Pérez Fraga, László Róbert Zsiros, Balázs Szigeti, Tamás Faragó

**Affiliations:** BARKS Lab, Department of Ethology, Eötvös Loránd University, Budapest, Hungary

**Keywords:** Dog, Speech, Social cognition, Vocal communication, Heterospecific communication, Ethorobotics

## Abstract

The concept of a “talking” dog has long fascinated humans, as presented throughout history in pieces of folklore, literature, and other fields of culture. While speech, as we know, is a uniquely human trait, the evolution of dogs in close proximity to humans has allowed them to develop strategies that facilitate heterospecific communication with us. In this work, we explore the scientific plausibility of enhancing canine vocalisation towards speech-like communication, as Csányi (Bukfenc és Jeromos: hogyan gondolkodnak a kutyák? Vince K, 2001) suggested. Our approach involves a comprehensive overview of the anatomical, cognitive, and evolutionary features of dogs that may relate to speech, as well as describing their role in popular culture and examining novel technological aspects. We also provide an outlook on hypothetical possibilities of a “talking” dog and its possible implications. We conclude that while dogs have acquired remarkable human-directed social and communicative skills, the feasibility and desirability of spoken language in dogs remain questionable. Instead, understanding canine vocal and non-vocal communication within the context of human–animal interaction provides valuable insights into both language evolution and the mechanisms underpinning interspecies cooperation, also providing practical tools for the novel field of ethorobotics.

“Let's make a talking dog!

[…] tens of thousands of years of domestication, mostly unconscious selection, have created a strange dog from the wolf, similar to humans in some ways and others. However, this is by no means the final option. The dog's mind has occasionally shown such high-level abilities that, with well-planned selection, we could produce dogs that are much more intelligent and communicate better than today's, even "talking" dogs. If we could breed more intelligent dogs that understand human speech and can express themselves better, it would be a joy for everyone, as we would have even kinder, more lovable and more empathetic friends. For example, how much would the value of a guide dog increase if it were not only to stop at a busy crossing but also signal with the word "car" or if its owner were looking for a phone booth, the dog would not only lead it there but also warn it with words? Assistance dogs for disabled people could also be more effective if dog intelligence, understanding and expression skills were further developed. We could also enjoy more family dogs if our companions were more intelligent, talkative, and understanding.” (Csányi 2001)

Language is one of the central features of human identity (Friederici 2017). While certain components of spoken language, such as vocal imitation and vocal learning abilities (Nowicki and Searcy 2014; Jarvis 2019), have been observed in other species (e.g. songbirds: Wilbrecht and Nottebohm 2003), fully developed language and the use of speech are uniquely human. It is no surprise, therefore, that understanding how it emerged and evolved has been one of the greatest quests across various scientific disciplines (Hauser et al. 2002). Many of these research projects have been framed within a comparative approach (Fitch 2000a) investigating which traits are necessary for language, especially spoken language, which ones are shared between humans and other species, and which are exclusive to humans. Among these species studied, one stands out for its close relationship with humans and shared ecological niche: the domestic dog (*Canis familiaris*).

Although the exact time and place of the appearance of human language and the dog domestication are both still unclear (Thalmann et al. 2013; Levinson and Holler 2014; Larson and Fuller 2014), dogs have indeed lived in the human linguistic environment for tens of thousands of years. It is easy to imagine that communicating verbally with humans, even if merely imitating human words, would have been so highly adaptive for them that, if possible, it would have already started to develop and spread very quickly. Are dogs really on the road to verbalisation? If not, the question arises: what is the reason? Which skills are necessary for speech production and comprehension abilities that the dog might possess, and which skills do dogs lack? What would it be like if it did happen? How would this affect the lives of dogs and humans? In this review, we explore the scientific plausibility of Csányi’s idea and overview the current literature aiming to integrate a wide range of ethological, neurobiological, linguistic, and philosophical approaches, but without diving too deep into specific topics, to describe where dogs might be on this suggested path and consider some ethical and social considerations of creating a “talking” dog.

## We want them to talk—A glimpse into a cultural phenomenon

The human fascination with language and the idea of whether animals can use it is deeply infused in folklore and popular culture. From Aesop's fables (Sax 2017) through other traditional tales from around the world (e.g. Knappert 1978; Nassau 2019) to modern literature and movies, stories often feature animals that speak, think, and, in general, act like humans. By anthropomorphising the animals (Korhonen 2019), these narratives conveyed moral lessons and teachings in both an engaging and easily understood way. Furthermore, they not only addressed human struggles but also explored the relationship between humans and nature (especially with animals), addressing the question of who we are in the world (Dunn 2011). If an animal stands out as the most frequently portrayed with anthropomorphic traits (including speech use), it is the dog (Taylor 2018 doctoral thesis; Włodarczyk et al. 2024). The evolutionary story of dogs, marked by their selection for cooperation and dependency on humans (Hare et al. 2002; Miklósi and Topál 2013a), along with the several functions that they have fulfilled (and still fulfil) in the human social world (Hare and Ferrans 2021), has given them a special place in the public imagination. Even today, stories of “talking” dogs—often representing values such as friendship, loyalty, and kindness—are found everywhere (e.g. film characters: *Scooby-Doo* (Gosnell 2002); *Bolt* (Williams and Howard 2008), or books: *Mo, the talking dog* (Booth 2013); *Smart dog* (Vande Velde 1998)*, Gaspode* (Pratchett 1990)). The take-home idea seems clear: humans have a lasting fascination with dogs that can speak.

Thereby, throughout history, there have been many examples like mediaeval performances featuring dogs “behaving as humans” and even “speaking” for human entertainment, with a notable increase in the eighteenth century (coinciding with the rise of pet keeping) and in the nineteenth century (see an overview in Włodarczyk et al. 2024). This long line of anecdotes and cases of dogs allegedly able to speak attracted the interest of not only laypeople but also researchers of the time. For example, in 1912, in the pages of Science, Harry Miles Johnson reviewed the report of Don, a “talking” dog from Germany (Johnson 1912), written by none other than Oskar Pfungst, who was already famous for debunking the counting horse, Clever Hans (Johnson 1912). Through clever experimentation and even recording and playback using phonographs, he concluded that this particular dog produced vocal sounds that merely induced the listeners to have an illusion of hearing speech.

Still, our fascination with the idea of being able to talk with our closest companion has followed us into more modern times: with recent technological advancements, various equipment and software solutions have emerged to offer (variably realistic) aids for opening communication channels between dogs and humans. Some, using smart collars (e.g. https://laica.io/), even wireless EEG devices (e.g. a failed project: https://www.indiegogo.com/projects/no-more-woof#/) and mobile phone applications (a recent overview: https://whitelabelfox.com/pet-dog-translator-apps/), claim to provide insights into the dogs’ minds, potentially opening a one-way communication channel by translating their vocalisations into human terms. Others are claimed to be a new, two-way communication channel: soundboards with buttons that play pre-recorded words, serving as a means of conversation between companion animals (mainly dogs) and their owners. On the owners’ side, these so-called Augmentative Interspecies Communication (AIC) devices complement verbal communication and are used to initiate interactions (Bastos et al. 2024a). On the dogs’ side, owners claim that their dogs use the buttons as a “speaking device” requesting activities and objects, combining them to form sentence-like structures, and even to express feelings or describe dreams (https://youtu.be/kQ2btFzDxPs).

One research group teamed up with the largest manufacturer of such AIC devices and published results pointing to the possibility that dogs’ button-presses may be deliberate communication attempts (Bastos and Rossano 2023; Bastos et al. 2024b). Meanwhile, other researchers express concerns about the anthropomorphic interpretation of button pushes as speech, especially considering that the level of spectral distortion of the recorded and played back words might hinder dogs from perceiving these sounds as the actual words they were meant to be (Higaki et al. 2025). Also, relying on such devices instead of paying attention to the dogs’ natural communication channels can be considered a questionable approach (Włodarczyk et al. 2024). Although these AIC devices might provide a tempting and relatively easy way of communication, they can potentially drive further the already progressing infantilisation of dogs (Blouin 2013; Kubinyi 2025) by making owners perceive their dogs as forming pre-grammatical sentence-like structures, similar to infants in the early stages of language acquisition. Such infantilisation in the long run might have serious disruptive effects on natural behaviours, emotional development, and stress-coping mechanisms, leading to serious welfare consequences.

Nevertheless, the charm of modern “talking” dogs does not necessarily require devices and IT solutions (Włodarczyk et al. 2024). With the rise of social media and easy-to-get smartphones, entertainers offering to experience the wonders of a “talking” dog have moved from circuses and village markets to the virtual space. Owners’ recordings of their dogs emitting speech-like sounds often go viral, much like the “button dogs”, and are featured in talk shows and collections of entertaining videos, sometimes even bringing financial benefits, just as in the olden days. In these videos, we can see and hear dogs engaged in interaction with their owners while producing speech-like sounds or, just as commonly, producing words and sentences sounding like “mama”, “I love you”, or similar utterances (Table 1.). While the former cases seem like results of spontaneous dog-owner interactions, the latter are most likely reinforced behaviours that emerged naturally due to unintentional positive feedback or through direct training.

**Table 1:** A list of online videos showcasing dogs as they produce sounds, which are likely interpreted as speech by the owner. The table contains the emitted speech-like sound, the owner's reaction, the breed of the dog(s) in the video, and the link to the video

Speech-like sounds	Owner's reaction	Breed	Link
“I love you ”	Owner gives treat and/or praises	Multiple	https://youtube.com/shorts/vH3nQgjUy8Y?si=zFNG_4H9KkR-zpFk
“Mama”; “I love you”	Owner gives treat and praises	French bulldog	https://youtu.be/HrO6LbXLu_I?si=30ykF_7i4VE15lB2
“I love you”	Owner praises	Husky	https://youtu.be/qXo3NFqkaRM?si=Edu6GQYjLSJWJ3Af
“I love you”	Training video for "I love you"	Husky	https://youtu.be/ip1c1UQigM8?si=-19O2GVwxW9sCjf8
“Mama”	Owner praises (?)	Labrador	https://youtu.be/uco9I5noLpY?si=b9DkKm0N8Edg8TuS
“Mama”	Owner praises	Australian cattle dog	https://www.youtube.com/shorts/ysi2SseVsBg?feature=share
“Mama”	Owner presents food	Australian shepherd	https://youtu.be/I_zW6APE1qQ?si=Ye816MqTnH-Zu-GO
“Hello”	Owner laughs	Coonhound	https://youtube.com/shorts/rrGP0O24T9Q?si=0t1r_zzjqX7Ua-s6
“Luna”; “I love my mom and dad”	Owner praises	American Staffordshire terrier	https://youtube.com/shorts/U2MkGrfR_g0?si=ds5bsA6tr8CPdRkk
“WOW”; “I want to go for a walk”	Owner praises	American Staffordshire terrier	https://youtube.com/shorts/BlE8veSWsRs?si=qZlkjOtteIvo0ToR
“Oh my God”; “I love my brother”; “I'm a good girl”	Owner praises	American Staffordshire terrier	https://youtube.com/shorts/ONk_xoXSoCc?si=9wHTAAzyPFlrC__K
Italian “accent”	Actively talking	Husky	https://www.youtube.com/shorts/GlDT8BFx1-Y?feature=share

Contrary to the potential clickbait titles and descriptions, just like Don in the previous century, these dogs do not talk either. Instead, they produce some natural elements of their repertoire (e.g. growl, moan, whine and howl, Faragó et al. 2014c), and categorical perception might trick us into perceiving these as speech. These sounds fall into the same spectral domain as human speech and have a similar harmonic structure, with marked frequency bands enhanced by the vocal tract, as well. These latter bands are called the formant frequencies and play a crucial role in human speech. Which frequency bands are enhanced or attenuated, and consequently, how far or close these fall to each other in the frequency spectrum, depends on the articulation (Fant 1960), differentiating vowels in human languages. In dog and, generally, non-human animal vocalisations, the position of the formant frequencies primarily depends on the shape and size of the vocal tract, but oral and laryngeal movements can dynamically modify their positions.

**Fig. 1:**
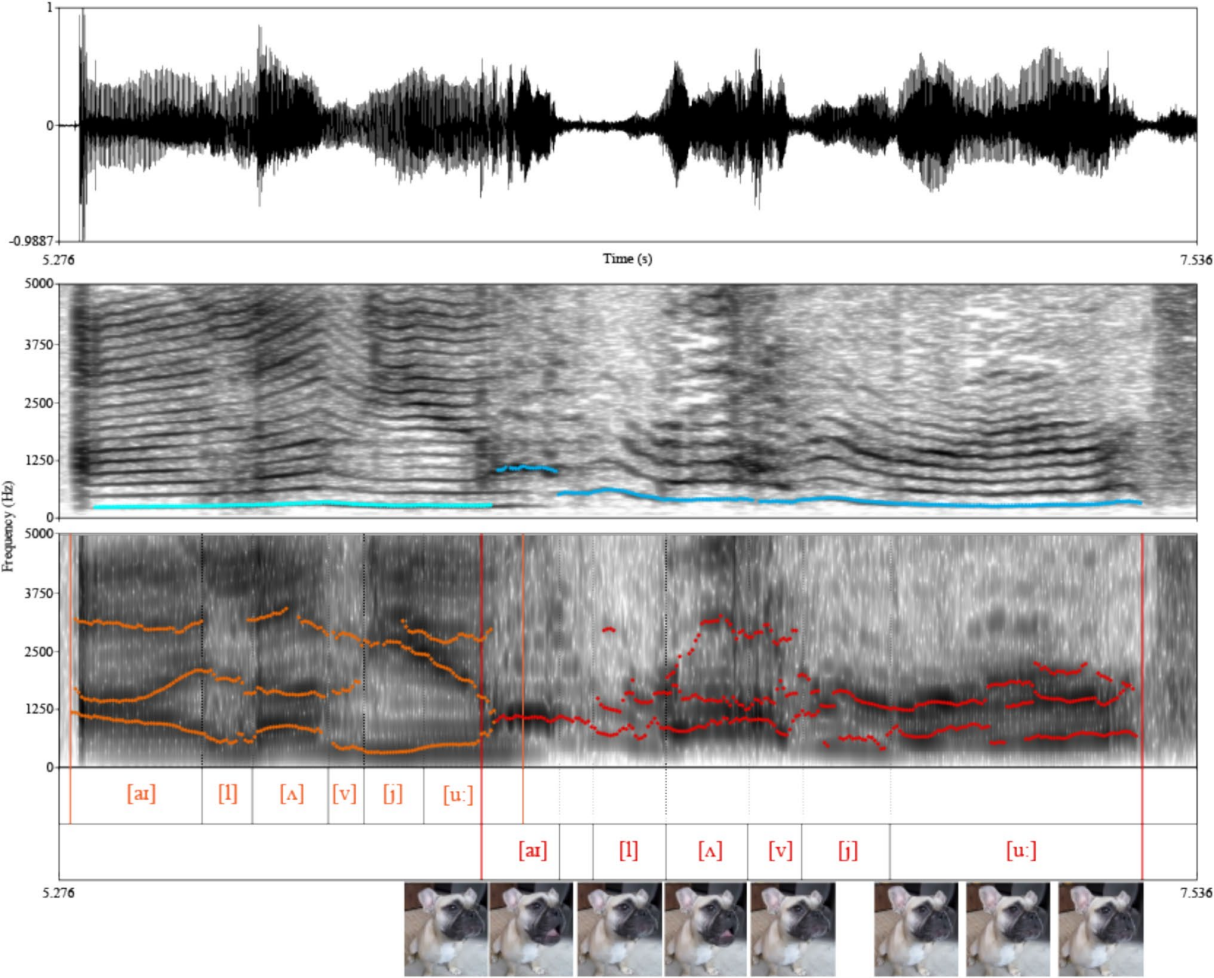
Sample from a “talking” dog video (https://youtu.be/HrO6LbXLu_I?si=DEPg6Xr72d6d_ogx&t=36) showing a French bulldog saying “I love you”. The top part shows the oscillogram representing the raw waveform of the sound; the middle is a sonogram showing the change of the sound’s power-spectrum over time optimised for frequency resolution (the greyscale colouring shows the power of a given frequency component: the darker a spot, the higher the power is), with highlighted pitch contours (cyan: female human, blue: dog utterances); the bottom is also a sonogram optimised for highlighting the formant structure (only the first three formants are highlighted; orange: female human, red: dog utterances). Note the similar height of the fundamental frequency (on average 275 Hz for the human and for the dog 431 Hz including, while 368 Hz excluding the high-pitched initial call), the similar harmonic structure (energy loss in the above 3000 Hz in the spectrum of the dog vocalisation is probably due to the distance difference from the microphone). While consonants are practically missing from the dog's utterance, vowel-like parts are similar to human speech sounds. The dog’s “I” [aɪ] sound is a bark-like short call; its fundamental and first formant falls in the same area as the human “I” [aɪ]’s first two formants. In the case of the “o” [ʌ] sound, the spectral similarity is remarkable. However, the closing “ou” [u:]’s formant structure differs greatly from the human version

Our brains, on the other hand, are heavily tuned to processing speech sounds (Vouloumanos et al. 2010; Chan et al. 2014; Riecke et al. 2018) and, due to categorical perception, readily interpret human-like formant configurations as vowels. Simply put, the phenomenon of categorical perception occurs when our brain creates distinct, non-overlapping categories and forces a perceived signal into one or the other, even if it actually falls between them (Goldstone and Hendrickson 2010). Additionally, there is no (or minimal) distinction between elements within a category, even if they fall far apart in reality. Consequently, although formant positions can change continuously, creating transient forms between categories in the case of speech sounds, specific formant structures are perceived as particular vowels even when their structure varies. In contrast, other formant structures are perceived as different, distinct vowels; however, upon hearing these intermediate structures, we still perceive them as that particular vowel that falls closer structurally. This phenomenon can also result in the illusion of speech sounds when hearing dog vocalisations (and might also be the basis of onomatopoeia). At the same time, mouth and lip movements, by stopping or modifying airflow and adding noisy elements to the sound, can create the acoustic illusion of consonant production. As a result, a sequence of such sounds becomes speech in the ears of the beholder (Fig. 1).

## What is the reality?—Vocal, neural, and social characteristics

Speech is a highly complex process involving the orchestration of sophisticated neural and biomechanical processes for production, but it also requires several abilities from seemingly unrelated cognitive domains. In the following sections, we will provide an overview, based on the latest findings, of the extent to which dogs possess these abilities, as well as the capabilities they may lack.

Two commonly mentioned pre-adaptations for the use of speech are the lowered position of the larynx, which statically elongates the vocal tract, and the enhanced laryngeal flexibility allowing dynamic modifications of the vocal tract (Hauser et al. 2002; Colbert-White et al. 2014). For a long time, the static lowering of the larynx was thought to be a uniquely human trait and was used to determine our ancestors’ speech readiness as well (Lieberman and Crelin 1972). However, recent studies suggested that not just a wider range of our antecedents (Boë et al. 2002; Clark and Henneberg 2017) but even rhesus macaque vocal tracts without this static lowering can produce speech-like vocalisations with spectral structures similar to our vowel sounds, lessening the importance of such static anatomical adaptations in speech-readiness (Fitch et al. 2016). Furthermore, recent research suggests that flexible larynx positioning in itself is also not as strict a prerequisite for speech-like vocalisations as once believed. Colbert-White et al. (2014) pointed this out in their comparative assessment of humans, apes, songbirds, and parrots—all capable of producing complex vocalisations. Moreover, human infants can also produce certain speech sounds before developing precise laryngeal control. In fact, by around 12 weeks of age, they begin to produce distinct vowel categories despite their anatomical and motor limitations (Kuhl and Meltzoff 1996). Still, little is known about the control of vocal organs in a wider range of non-human mammals, including the active lowering and flexible positioning of the larynx. Some cineradiographic observations of vocalising dogs (among a handful of other studied species) show dynamic larynx movements. This ability to actively lower the larynx would be enough to allow dogs to produce a wide variety of formant frequencies (Fitch 2000b; Fitch and Reby 2001). Moreover, a shorter head has been reported to be associated with a more pronounced static lowering of the larynx too (Plotsky et al. 2016). These findings, taken together, may suggest that greater vocal flexibility was gained during domestication, particularly in dog breeds with pronounced brachycephaly (Lesch and Fitch 2024). All in all, these would suggest that dogs could still exhibit sufficient vocal flexibility to produce speech-like sounds.

Indeed, dogs exhibit remarkable vocal variability, in line with the reports of their flexible vocal apparatus. It was found that they modulate their voice context-specifically (Faragó et al. 2010b; Bálint et al. 2013, 2016), the acoustics of their growls contain indexical (e.g. body size), contextual (e.g. food guarding vs repelling threat), and inner state (e.g. aggression, fear or joy) information for others (Taylor et al. 2009; Bálint et al. 2013; Faragó et al. 2017; Pongrácz et al. 2024), and they also perceive and react to changes in the formant frequencies, extracting size cues of conspecifics (Faragó et al. 2010a; Taylor et al. 2010, 2011). Also, dogs show certain vocal flexibility: canids, including dogs, were described to use call combinations (e.g. bark-howls) (Cohen and Fox 1976) and transient intermediate forms of calls (e.g. the moan that is acoustically between growls and whines, Schassburger 1993). They also appear to have some control over their vocal apparatus, although there is no evidence that wild canids are advanced vocal learners. There are indications that dogs can learn to vocalise on command, as shown in the 1960s (Salzinger and Waller 1962). It is also suggested by the fact that teaching dogs to vocalise on command is a popular training trick, as well as the long line of historical examples of “talking” dogs. Furthermore, there is some evidence, although (so far) very limited, of vocal imitative abilities present in dogs (Topál et al. 2006).

Over the last 20 years, several aspects of dogs’ vocal behaviour have been studied (e.g. growls: Taylor et al. 2009; Faragó et al. 2010b, whines: Marx et al. 2021a, 1b, , and howls: Lehoczki et al. 2023). Still, our overall knowledge about their vocal repertoire is surprisingly limited, based on outdated methodology (Bleicher 1963) and mainly on wolves' repertoire (Schassburger 1993; Faragó et al. 2014c). What is certain is that domestication, possibly through its effects on neural crest development, impacting both laryngeal morphology and neural control (Lesch and Fitch 2024), has significantly changed dogs’ vocal behaviour. In the famous selection experiment on farm foxes (*Vulpes vulpes;* Trut 1999), researchers found that traits associated with domestication syndrome emerged as byproducts of artificial selection for tolerating human proximity and reduced aggression against humans (Trut 1999; Trut et al. 2009). Interestingly, the vocal behaviour of these foxes also changed dramatically, particularly the usage rate and context of usage of certain call types (Gogoleva et al. 2009, 2010). Dogs were the first domesticated animals (Larson et al. 2012), and the domestication process certainly involved early selection for tameness, which may have affected their vocal behaviour (Pongrácz 2017), as demonstrated by the farm fox experiments. Humans, as an extremely alloparenting species, show numerous examples of raising heterospecific "offspring", not only in Indigenous tribes but also among modern, urban-living people (Serpell 2021). Likely, 30,000 years ago, from the ancestral dogs that were adopted and hand-raised, individuals that reacted better to vocalisation-centred human communication during their upbringing were favoured and consequently reached maturity and reproduction more likely. This pressure might have also favoured individuals with greater neural and vocal flexibility and sensitivity to human pedagogical behaviour (Topál et al. 2010). Accordingly, the most studied dog vocalisation, the bark, was shown to have expanded significantly during domestication (Pongrácz et al. 2010; Pongrácz 2017) and became more prevalent in the vocal repertoire than it can be seen in wild canids, and also likely in their ancestral form, developing into an important channel in dog–human communication. As a parallel, possibly complementary process, howling, a central call type in the canid communication system, degraded and lost its original communicative function in dogs (Lehoczki et al. 2023).

Regarding perception and speech processing abilities, there are indications that dogs can form mental representations of objects and associate these with sounds, suggesting the presence of auditory learning in a surprisingly advanced way. Recent neural findings suggest that family dogs have at least a visual mental representation of known objects referred to by their names (Boros et al. 2024) and also have multisensory representations, which can be recalled by verbal labels (Dror et al. 2022). On a lower level, dogs seem to process voice-like sounds similar to humans (Bálint et al. 2023) and are sensitive to specific infant and dog-directed prosodic cues (Gergely et al. 2023). Furthermore, dogs seem to be able to differentiate languages (Cuaya et al. 2022) recognise their owner (Gábor et al. 2022) or familiar persons (Surányi et al. 2024) based on voice only and show evidence of statistical learning of lexical information (Boros et al. 2021). They can also differentiate between two learned tones in a discrimination task (Starling et al. 2014) or associate sounds with spatially oriented motor responses (Gergely et al. 2014). Notably, another key feature relevant to speech perception is hemispheric asymmetry, which refers to specialised brain organisation in one hemisphere for processing vocalisation (Bradshaw and Rogers 1993; Belin et al. 1998; Peelle 2012). Although initially thought to be unique to humans, lateralised processing of vocalisations has also been identified in apes, parrots, and songbirds (Colbert-White et al. 2014). In dogs, an fMRI study found a right-hemisphere bias when processing meaningful words compared to non-meaningful ones (Andics et al., 2016), much like humans. In other studies, no lateralisation was found, for example, concerning human language representation in the canine brain (Cuaya et al. 2022).

In addition to the changes in the neural background and vocal communication, there are several socio-cognitive skills whose emergence is thought to have served as a prerequisite for the later development of language and speech in humans (Levinson 2006). Sociality, including frequent conspecific interaction, individual recognition, and extensive parental care, is thought to lead to the development of diverse communicative signals and complex vocal repertoires, as seen in apes, parrots, and songbirds, in which speech-like vocalisations have been found (Colbert-White et al. 2014). However, little is known about the extent to which these features are truly human-specific or to what extent it was a unique, lucky combination of our evolutionary heritage and/or environmental factors that made the development of verbal communication possible (Heesen and Fröhlich 2022). According to Levinson's hypothesis, the primary driving force behind the development of these abilities may have been the need for cooperation and the coordination of complex joint actions (Levinson 2019). Even in the everyday lives of companion dog owners, it is often observed that dogs are able and willing to cooperate with humans in complex ways. This cooperativity is partly the result of the social behaviour inherited from the common ancestor with the grey wolf (*Canis lupus*) and partly may have evolved due to living in the same ecological niche as us (Range and Virányi 2014).

The exhaustive exploration of all the necessary features for language evolution is an ongoing process. This communicational and cognitive skillset can be described along four main domains that seem to be universal to any human interactions: (1) face-to-face multimodal communication: communication through different sensory channels; (2) communicative turn-taking: rapid exchange of communicational turns; (3) sequence organisation: communicative contexts that are contextually linked to preceding and following acts; and (4) intentionality: the ability to engage and respond to the other’s intentions (Heesen and Fröhlich 2022). Originally considered human-specific, these elements or their components have also been found in non-human species (Abreu and Pika 2022), and it is still an interesting proposition to examine dog behaviour from this aspect, too. Mainly because, in contrast to primates (Jack et al. 2008), diverse interactions with humans are an inherent part of dogs’ natural behavioural repertoire (Topál et al. 2009). For instance, one of the main preconditions of face-to-face communication (1) is the disappearance of gaze aversion, which can be markedly observed in dogs (Soproni et al. 2001; Téglás et al. 2012; Wallis et al. 2015; Duranton et al. 2017). Multimodality itself is also present even in their intraspecific behaviour (Déaux et al. 2015), while they also process signals from humans both through visual and auditory channels (Scandurra et al. 2020). Regarding the ability of turn-taking (2) in general and sequence organisation (3), the most prominent example might be the work of guide dogs, which requires a high degree of behaviour synchronisation. Naderi et al. (2001) investigated which half of the dog-blind person dyad initiates the actions during a regular walk. They found that this joint action between the dog and its owner can be described as an action sequence where the members take turns, and the role of the initiator rapidly changes. Moreover, when investigating how naïve pet dogs perform, they found that they were innately prone to cooperate with their owners, without any specific training (Naderi et al. 2001). Dogs also react sensitively to the attention state of humans (Gácsi et al. 2004); they can distinguish intentional and unintentional (4) actions (Schünemann et al. 2021), and their “showing behaviour” towards a hidden reward is considered to be functionally referential, which could also be an indicator of intentionality (Miklósi et al. 2000). Moreover, it was found that they show signs of joint intentionality with humans, as upon interruption of a social play session, they try to re-engage with their former partner over an equally familiar but previously passive person (Byrne et al. 2023).

We have reviewed that dogs indeed possess some abilities, at least to some extent, that language requires, such as vocalisation control, perceiving and processing auditory information, and engaging in communicative exchanges. However, it is evident that some other crucial aspects of verbalisation are absent from them, as they cannot speak. Beyond vocal signalling, language is a rule-governed system comprising multiple layers: phonology, morphology, syntax, and semantics (Kastovsky 1977; Zsiga 2024). These are not entirely without precedent among non-human animals (Suzuki et al. 2020), but mastering these elements requires additional cognitive capacities, such as event segmentation—the ability to perceive the continuous stream of sensory information as discrete, meaningful units (Zuberbühler and Bickel 2022). While dogs likely possess some degree of speech segmentation ability through statistical learning (Boros et al. 2021), whether they can engage in higher-order syntactic processing, to what extent, and how it compares to human syntax remains an open question.

Furthermore, we must also examine speech and language within the context of their primary mode of use in human social interactions: conversations. In human societies, speakers must avoid interrupting or overlapping with each other during a dialogue. However, the neurological and cognitive mechanisms underlying rapid communicative turn-taking are far from trivial. The average gap between turns in human conversation is approximately 200 ms—far shorter than the time required to plan and articulate a response (Levinson and Torreira 2015). This swiftness suggests that speakers must predict the end of the current turn and simultaneously formulate their own utterances while still processing the ongoing speech (Bögels and Levinson 2017). The ability to turn-taking itself is not unique to humans, as coordinated vocal exchanges have been observed across a range of vertebrate taxa, including duetting songbirds (Brenowitz 2021), great apes (Pougnault et al. 2022), meerkats (Demartsev et al. 2018), and dolphins (Moore et al. 2020). As previously noted, canines, too, demonstrate social coordination and some form of turn-taking in various contexts (Naderi et al. 2001; Bauer and Smuts 2007; Nilsson 2020). However, humans possess an additional capacity, the ability to engage in multiple parallel conversational threads within a single interaction. Humans dynamically manage airtime (the time a speaker talks), turn-taking, and backchannel feedback across numerous participants with remarkable precision—an ability not yet observed in any other species (Cooney et al. 2020).

To summarise, language as a referential, complex, and flexible communication system relies on multiple interdependent factors. The existing literature suggests that many of these putative prerequisites for speech are present to some degree in certain non-human animals, including dogs. Yet, despite these shared features, dogs have not developed human-like verbal communication. It suggests that some other key human anatomical and cognitive adaptations may have played a crucial role, in addition to the prerequisites reviewed here. Indeed, there are some theories that, by their nature, exclude the possibility of examining them in dogs. One such theory, the gesture-first hypothesis, proposes that our bipedal ancestors used their free upper limbs for gestural communication, providing a foundation for early language evolution (Steele et al. 2012). The dexterity afforded by our opposable thumbs may have also played a pivotal role: the hypothesis on tool-making and language co-evolution suggests that the cognitive demands of tool use and linguistic structuring developed together, reinforcing each other (Stout and Chaminade 2012; Morgan et al. 2015; Kulik et al. 2023). An interesting addition here is that there may be other crucial, unidentified elements—cognitive, developmental, or evolutionary—that preclude speech from emerging in dogs, which scientists have not yet identified as a factor in language development. However, based on our current knowledge, these cannot be tested or falsified (Popper 2005).

## Do they need to talk at all?—Interspecific communicative abilities

The idea of a “talking” dog that understands us and expresses itself better might seem tempting and innovative at first glance, but are not dogs already quite skilled at navigating our communicative world? Do they really need to talk for this? Indeed, although dogs lack the capacity for speech, it is widely acknowledged that they have developed outstanding human-directed communicative abilities (Hare et al. 2002). Such skills are believed to build upon already existing characteristics of dogs' ancestors, such as high cooperativity and gregariousness, a rich intraspecific communicative repertoire, and sensitivity to visual social signals (Cooper et al. 2003; Miklósi and Topál 2013a). Artificial selection by humans further shaped dogs' interspecific social skills to facilitate and enhance human–dog communication and cooperation (Hare et al. 2002; Gácsi et al. 2009c), as well as their fit in the anthropogenic niche.

For instance, dogs prefer to communicate with humans who have a visible face (Gácsi et al. 2004), and they readily use eye contact from puppyhood (Gácsi et al. 2005; Gerencsér et al. 2019). In human communication, the visibility of the face is key to recognising the other person’s attention, and eye contact is considered essential for establishing a proper communicative channel (Emery 2000). Dogs are also sensitive to the ostensive nature of this cue (Gácsi et al. 2005; Gerencsér et al. 2019), showing increased attentiveness and better performance in different tasks after establishing eye contact with humans (Virányi et al. 2004; Kaminski et al. 2012; Savalli et al. 2016; Duranton et al. 2017). Furthermore, dogs demonstrate a remarkable ability to interpret and use human gestural communication. Numerous studies have shown that dogs successfully locate hidden food rewards in several contexts by following different human pointing cues (Miklósi and Soproni 2006; Kaminski and Nitzschner 2013). Pointing is a gesture predominantly used in our communication (Liszkowski et al. 2012) and is considered quite human-specific (Leavens and Hopkins 1999; Miklósi and Soproni 2006). Additionally, dogs can also follow human gaze direction (Miklósi et al. 1998; Wallis et al. 2015; Catala et al. 2017). The inherent nature of dogs’ sensitivity to human communicative gestures is evident in the fact that juvenile dogs with minimal exposure to humans exhibit similar responses (Riedel et al. 2008; Gácsi et al. 2009b; Bray et al. 2021b).

Furthermore, dogs are not only attentive and able to interpret various human communicative signals, but they also display interspecific communicative behaviours themselves. In fact, dogs have been found to flexibly use their gazing behaviour as a form of human-directed communication (Cavalli et al. 2018). First, they might gaze at the humans, often accompanied by vocalisations and physical interactions (e.g. pawing, jumping) to beg or simply to grab the human’s attention (Gácsi et al. 2004; Gerencsér et al. 2019). When they face a difficult problem or an ambiguous stimulus, dogs look back at their human partner—a behaviour widely regarded as an attempt to initiate a communicative interaction (Miklósi et al. 2003; Marshall-Pescini et al. 2017) or to seek information from the human’s behavioural reaction to it (Merola et al. 2011). Although this communicative phenomenon is present in the general dog population, the artificial selection for different functions also seems to have modulated it, with the so-called cooperative breeds forming eye contact faster (Gácsi et al. 2009c; Bognár et al. 2021) and looking at their human partner longer and more frequently in problem-solving settings compared to independent breeds (Passalacqua et al. 2011; Pongrácz and Lugosi 2024)**.**

Additionally, dogs commonly use gaze alternations between a human and a desired target out of their reach as an attention-grabbing and directional behaviour (Miklósi et al. 2000; Savalli et al. 2014). Rapid gaze alternation has often been described as the benchmark behaviour of gestural functional referential communication in non-human animals (Malavasi and Huber 2016; McElligott et al. 2020; Zeng et al. 2024), with a similar function to human pointing gestures (Leavens et al. 2005; Marshall-Pescini et al. 2013; Savalli et al. 2014). In addition to gaze alternation, other proposed criteria must be met to establish referential communication (Leavens et al. 2005), such as the use of attention-getting behaviours, the presence of an audience whose attentional state is taken into account, and the persistence or even an elaboration of communicative behaviours when the initial attempts to influence the receiver fail (Leavens et al. 2005). Research suggests that dogs fulfil most of these criteria. For instance, when displaying gaze alternations, they consider the audience’s attentional state (Marshall-Pescini et al. 2013). They also frequently accompany gaze alternations with other attention-grabbing behaviours (Miklósi et al. 2000; Gaunet 2008), persist in their display (Gaunet 2010), and even there are some indications that they elaborate on these behaviours when the recipient does not respond (Savalli et al. 2014).

Furthermore, dogs and humans have been found to recognise each other's emotional expressions, a skill that is crucial for evaluating the social motivations of others within the group and responding accordingly (Schmidt and Cohn 2001). First, dogs are suggested to be skilled at reading human emotions, a statement not only made by their owners (Szánthó et al. 2017) but also supported by several studies. Dogs can discriminate human emotional vocalisations (Siniscalchi et al. 2018) and facial expressions (Müller et al. 2015), even adequately matching these two modalities (Albuquerque et al. 2016). Along with this, dogs seem to use the emotional information received from humans as they adjust their behaviour accordingly (Albuquerque and Resende 2023)—both in their responses to the human (Bräuer et al. 2024) and in using that emotional information to guide their own decision-making (Merola et al. 2011; Fugazza et al. 2018; Albuquerque et al. 2021). Signs of emotional contagion, an automatic inner state matching between the signaller and the receiver, which is suggested to allow information transfer and group coordination (Briefer 2018) were also found in dogs after hearing human emotional vocalisations (Yong and Ruffman 2014; Huber et al. 2017; Lehoczki et al. 2024), or after witnessing their owners experiencing a stressful event (Katayama et al. 2019). Additionally, some studies suggest that dogs respond in contextually appropriate ways during post-conflict interactions, for example, after being scolded by their owners, which constitutes an emotionally negative situation (Cavalli et al. 2016) and also engage in consolation behaviours when observing their owners in distress (Custance and Mayer 2012; Rial et al. 2024). On the other hand, dogs produce acoustically different barks (Pongrácz et al. 2005) and growls (Faragó et al. 2017) depending on the context when interacting with humans, which in turn, humans seem to categorise in both context and emotional content accurately. Moreover, humans associate emotional states with a wide range of dog vocalisations following similar rules as they apply to human vocal emotion expressions, too (Faragó et al. 2014a).

Research has also questioned whether the aforementioned human-oriented socio-communicative abilities are unique to dogs or a general result of domestication or learning through ontogeny from humans (Udell et al. 2009). Indeed, similar human-oriented communicative behaviours, as those observed in dogs, have been reported in other domestic animals, like horses (Malavasi and Huber 2016), goats (Kaminski et al. 2005; Nawroth et al. 2016a), pigs (Nawroth et al. 2016b), cats (Pongrácz et al. 2019; Zhang et al. 2021) and even in human-socialised wild species, like dolphins (Zeng et al. 2024), kangaroos (McElligott et al. 2020), and wolves (Virányi et al. 2008; Heberlein et al. 2016). While these results highlight the undeniable effects of domestication and socialisation on animals’ capacities to communicate with humans, they do not override the fact that dogs seem to be especially predisposed to engage in human-oriented communicative interactions. When directly compared to similarly socialised individuals of other species, dogs outperformed pigs (Gerencsér et al. 2019) and wolves (Gácsi et al. 2009a; Salomons et al. 2021) in responding to human-given cues, learning actions demonstrated by humans (Gácsi et al. 2009a; Fugazza et al. 2023) and in producing human-directed communicative behaviours (Miklósi et al. 2003, 2005; Marshall-Pescini et al. 2017; Pérez Fraga et al. 2021). Dogs also appear to be more attuned than other species to the emotional content of human vocalisations (Lehoczki et al. 2024). And notably, dogs exhibit many of these human-oriented behaviours with minimal experience with humans (Bray et al. 2021a). Even the propensity to display more complex behaviours, such as gaze alternations (which involve the production of communicative signals rather than merely comprehension), emerges at a young age and is consistently observed across various contexts and scenarios (Passalacqua et al. 2011; Gaunet and Deputte 2011; Pérez Fraga et al. 2021).

## Look who's talking—possibilities of a hypothetical experiment

We described how humans are fascinated by the concept of a “talking” dog, as we tend to attribute all the human virtues to them. We also argued that dogs have already developed several skills to understand us and make us understand them. Still, for the sake of a thought experiment, let us imagine the consequences if dogs mastered human language. Importantly, our aim here is not to have a comprehensive review of: (1) the plausibility of the development of a “talking” dog; (2) the details of the changes required; (3) the potential cognitive and behavioural effects; (4) what dogs might express; or (5) the possible positive and negative impacts on both dogs and humans. Instead, we offer just a few, but as broad as possible, food-for-thought examples of the utopian (or dystopian) consequences of creating a “talking” dog.

First of all, we need to discuss what a “talking” dog is. The first and most possible scenario is that, due to some changes in their vocal apparatus—a result of artificial selection—they would be able to produce more sounds that humans recognise as words. Moans are one of the best candidates to become such speech-sounding calls. Their pitch, although it can vary in a wide range, overlaps with the human speech register (80–600 Hz); they are relatively tonal and are used in emotionally ambiguous contexts (Faragó et al. 2014c). In such contexts, interesting and salient patterns may more likely evoke the needed attention, thus leading to the owners' unintentional reinforcement, which in turn will elevate the occurrence of these peculiar calls. Then, building on these precursor sounds, more direct training can further shape them into the desired speech-like sounds that the dogs can produce on command. Such scenarios are indeed possible, as previously demonstrated by the videos in Table 1 and the French bulldog shown in Fig. 1. However, the rarity and uniqueness of these examples, as well as the limited range of uttered speech-like sounds, suggest that the above-mentioned vocal apparatus changes are indeed required for more elaborate speech.

Then, they could spontaneously associate or be taught to name objects or actions through conditional learning. Imagine a dog that, when attempting to get the attention of its owner because it wants to go out, produces a sound that sounds like the word “walk”. The owner certainly will react to such a coincidence, and their reaction will reinforce the behaviour. However, if this involves not just attention but an actual walk, the dog’s brain might form an association between the produced call and the action of going out for a walk. Research suggests that dogs may be more predisposed to learn verbal cues of actions rather than objects (Ramos and Mills 2019). Thus, they would probably also associate the articulation of these words more easily. Meanwhile, some so-called gifted dogs show an exceptional ability to learn object names (Ramos and Ades 2012; Fugazza et al. 2021a, b) and can recall these names even in the long term (Dror et al. 2021, 2024), but most dogs show only limited capacity for this skill. Naturally, existing research on canine vocabulary learning primarily focuses on their receptive vocabulary (Dror et al. 2021, 2024; Fugazza et al. 2021a). Thus, it is an interesting question, to what extent that skill would translate into productive vocabulary. In the above example, if the dog starts using the call to directly “request” walks outside the original context where the association was formed, that might suggest such a precursor of speech production. However, it is important to emphasise that this level of word production is still merely a result of conditional learning rather than true language production. Here, we might consider research on Alex, the Grey parrot, as evidence that non-human animals can develop communicative abilities with human-like characteristics, including the capacity for meaningful two-way interactions and some understanding of concepts such as numbers and object permanence (Pepperberg 2006). However, even Alex’s abilities remained very limited compared to those of complete human linguistic competence. Similarly, a “talking” dog would likely have constraints in its ability to form complex, novel expressions beyond what it learned.

In most cases, non-human species communicate their inner states to influence others' behaviour (Rendall et al. 2009). However, as we mentioned, one of these exceptional cases is the dog's gazing behaviour itself, which they use to communicate with humans referentially (Miklósi et al. 2000). Still, even primates taught to use sign language or other devices to communicate primarily expressed their own needs (Tomasello 2016), so we can assume that it would be no different in the case of a dog. Thus, another scenario of a talking dog is that they might communicate in a way dogs do in their natural environment, but use human-like words to express themselves and use them alongside or instead of their natural signals. However, if dogs could communicate this way, it would have to be accompanied by not only the ability to separate their affective state from their own communicational signals and use symbols instead of them (Olney 2013). But also, it would assume an ability of self-perception and awareness of the dog’s own emotional state (Salzen 1998; Mendl et al. 2022), which would undoubtedly require more capacity, which has not been proven to date and that might require greater alterations than the ability to associate word-like sound sequences with objects or actions. Even human children begin to communicate about their mental states late in their second year of life, but this becomes more prevalent during the third year (Bretherton and Beeghly 1982).

At the highest complexity we cannot even conceive, but we must still mention, if dogs were able to achieve fully human-like communication, using structured language with syntax, abstraction, and flexible expression, which goes far beyond naming objects or signalling needs. However, changing from a dog’s current state to a human-like linguistic level would represent a drastic transformation. Even in humans, there is a theory about how language shapes thinking, despite all humans sharing a common linguistic ability (Wolff and Holmes 2011). This phenomenon is typically observed in nuanced domains such as colour perception, spatial references, and number (Wolff and Holmes 2011) or time representation (Boroditsky 2001). If dogs were to develop human-like language abilities, this could also mean that their cognition and behaviour would be so dramatically altered that we might consider them as a distinct species from the present-day ones, just like in the case of grey wolves and dogs, where the behaviour of the dog has changed so significantly due to domestication (Miklósi and Topál 2013b), that we handle it as a separate species, though they are able to reproduce with the wolf (Vilà and Wayne 1999).

## Are we talking up the wrong tree?—Implications for dogs and humans

At first sight, the idea of having a dog that can speak and understand our language (even if we imagine the first and most plausible version, where the dogs can produce only a limited number of words) seems advantageous in various aspects: for their efficiency in different working roles alongside humans and better communication when they are kept as companions, ultimately increasing the dogs’ quality of life. For instance, working dogs that cannot see their handler’s face often display behaviours associated with seeking additional information (Bryant et al. 2018). Thus, improving human signalling could enhance their performance in these situations. Furthermore, dogs with a better comprehension of human gestures tend to be more successful as assistance and detection dogs (MacLean and Hare 2018). Therefore, their performance in these roles and situations could improve if they could better understand our language and respond accordingly. Even a limited vocabulary may provide real benefits for service dogs, enabling them to communicate key information more accurately (e.g. guide dogs warning of specific obstacles, detection dogs verbally identifying goods they have found, rescue dogs assuring victims that help is on its way, etc.).

We can imagine a similar scenario in the household environment. Although we have described above that dogs are attuned to human communicative channels and can make themselves understood, there are still some situations in which humans misinterpret dogs' signals, which can pose a potential risk to humans, such as stress-related ones (Demirbas et al. 2016; Meints et al. 2018). Therefore, in these contexts, having a dog which can express its inner states or at least say some words about the current situation would be undoubtedly advantageous. However, this is not limited to extreme contexts. We must also consider that dogs are deeply human-oriented, with their relationship to the owner being analogous to the parent–child attachment bond (Topál et al. 1998). We could easily imagine a dog expressing a preference for its owner over others, showing specific behaviour patterns upon reunion, and communicating its need for its owner in uncertain situations—all through language. Indeed, such loquaciousness could further strengthen their bond. Likewise, dogs would not lose their “dogness”, continuing to respond to human emotions and accompanying their owners in various activities where they could utilise this new skill, while still showing affection and happiness through words. Together with this, thinking on the other side of the leash, humans care about their dogs and strive to ensure their happiness and well-being (Greenebaum 2004; Schaffer 2009). Indeed, recent years have seen growing awareness about the importance of positive welfare (Rault et al. 2025) for animals under our care, with research focused on identifying species-specific indicators of positive emotions and exploring the complex issue of sentience—the conscious experience of emotional states (Briefer 2020). One might imagine that teaching dogs to speak could offer a shortcut to understanding their inner states, allowing us to ask them directly about their experiences, health, and feelings (see some examples among the button dog videos). This cut-off could potentially enhance their welfare by providing more precise insights into their emotional and physical well-being.

However, another possibility is that the advantage of speaking could quickly turn into a disadvantage. First of all, regarding dogs’ welfare, although it is surrounded by lively debate, testing animals is still a current practice, for instance, in the field of biomedical research (Petetta and Ciccocioppo 2021). If a dog could easily answer how it feels and what symptoms it experiences, it could quickly become the most popular subject of human medical or even cosmetic research, despite advances in animal welfare and the push to develop substitute methods (Silva and Tamburic 2022). For example, in the case of medicine for the treatment of depression, researchers would not have to rely only on the results of often lengthy and complex behavioural tests or other more invasive methods, which can only indirectly measure the subject's affective state nonetheless (Belovicova et al. 2017).

Furthermore, dogs are dependent on humans, even free-roaming dogs are (Pingle 2024), but in the case of companion animals, it is more definite as the fulfilment of all of their essential needs depends on their owners (Meyer et al. 2022). Indeed, they not only express affection towards their owners throughout the day but also beg for food, complain when left alone, locked in an apartment, or simply seek their owner’s attention. Therefore, they often experience negative inner states during their everyday life, such as frustration (Lenkei et al. 2021). They constantly express these emotions, particularly through vocalisations. People can determine the emotional content of these vocalisations, especially barks, with certain bark types affecting them more disturbingly than others (Jégh-Czinege et al. 2019). Additionally, there are indications that dogs’ whines have similar acoustic parameters to children’s cries and also elicit caring behaviour (Lingle et al. 2012; Massenet et al. 2022). Although we did not find a direct comparison of whether dog whining or speech can be more annoying, it is known to what extent continuous speech in the background is distracting, even if it is not directly addressed to someone, like the background speech in an office. It is also known, for instance, that it has a negative impact on cognitive functioning (Schlittmeier and Liebl 2015). Thus, listening to "*I'm hungry, I'm hungry*" for several minutes might have a different effect than gazing or silent whining, which may still be easier to ignore (Archer 1997)—if somebody wants to. This effect could result in those behaviours that could otherwise be considered neutral; for instance, the dog sits next to a closed door where it wants to go out, might become demanding or annoying for the owners if they were verbally expressed.

There are many reasons why people keep dogs, but one of the most frequently reported is to have companionship (Holland et al. 2022). People talk to their animals, share their joys and sorrows with them, and treat them as family members or even as child substitutes (Greenebaum 2004). It is also very common for young couples to get a dog before the birth of their child or to get a dog after their own children leave the family home (Wise and Kushman 1984). From many aspects, dogs can fulfil the function of human social relations (Basten 2009). They might have a similar function in the family, but it is still much less demanding than raising children or having any kind of social relationship with a human partner. Naturally, there might be countless reasons for this, but one of them is undoubtedly the lack of verbality. One of the big "advantages" of the dog, compared to a human social partner, is that if we don't feel like it, we can simply ignore them, without having to worry about them, and what is more important: they do not talk back (Archer 1997). However, this asymmetric dynamic would be greatly changed if the dog could speak. The aspect of unconditional positive regard that often makes people favour their animals over humans (Aumer et al. 2022) might disappear.

Furthermore, as we have already stated, even if dogs were physically capable of forming some human words, this would not necessarily imply any change in their cognitive abilities. Here, the danger lies in the fact that people are already inclined to anthropomorphise their dogs, which has some positive effects (e.g. anthropomorphistic description and framing of dogs could promote a more supportive attitude towards them and facilitate their adoption; Butterfield et al. 2012) but also considerable negative consequences regarding the welfare of the dog. For example feeding them with inappropriate human food as an act of affection can lead to obesity or other severe problems; dressing them with inappropriate clothing to have a cute/fashionable look can impair their ability to thermoregulate and express natural behaviours; carrying them in the arms or bags could limit experiences with social and environmental stimuli hindering their cognitive and emotional development, also preventing them to work out coping strategies for those stimuli (Mota-Rojas et al. 2021), which would likely be even more pronounced in the case of a “talking” dog. Even now, when most internet users are familiar with Large Language Model (LLM)-based AI systems like ChatGPT, we see how human-like conversational abilities can blur the line between artificial and natural intelligence, raising expectations beyond what the system is actually capable of (Abercrombie et al. 2023; Ferrario et al. 2024). This anthropomorphisation parallels the potential consequences of creating “talking” dogs, meaning that if a dog could articulate words, people might overestimate its cognitive abilities, attributing human-like reasoning where none exists. Just as passing the Turing Test does not equate to proper understanding (Turing 1980; Saygin et al. 2000), a "talking" dog might simply be producing learned vocalisations without genuine linguistic comprehension. The risk is that such illusions could distort our perception of animal cognition, leading to unrealistic expectations and ethical concerns about how we treat these animals (see Włodarczyk et al. 2024 for a similar concern about button dogs).

Producing speech by dogs opens the door to a different—rather worrying—perspective, too: the uncanny valley. While this concept was first described in the context of robots by Masahiro Mori in 1970 (Mori 1970, 2012), this phenomenon can extend beyond humanoid machines to any entity that violates deeply ingrained expectations in us, evoking a feeling of unease (Kätsyri et al. 2015). There are various potential evolutionary explanations for what biological processes might be behind this uncanny valley, from disease (Curtis et al. 2011) or threat avoidance to perceptual mismatch effects (Kätsyri et al. 2015), all suggesting the plausibility of living entities being potential triggers too. Just as robots with near-human but imperfect features can appear unsettling, dogs producing speech-like sounds may provoke a similar avoidance reaction due to perceptual mismatch as they breach our intuitive boundaries of what is natural in canine communication. According to ethorobotics, in robots, especially social robots that are required to operate in close proximity with humans and engage in regular interaction with them, the embodiment should determine their socio-cognitive and communicative abilities (Miklósi et al. 2017). This approach means that, for example, while a humanoid robot can be expected to speak, a robot with a simple, non-human form should use other, simpler methods of vocal expression to be perceived as more acceptable (see the duo of C-3PO and R2-D2). This principle is rooted in the same biological processes that are thought to be behind the uncanny valley effect (Steckenfinger and Ghazanfar 2009). Just as non-humanoid robots should not communicate with speech as they would be perceived to be repellent, dogs should not either. The opposite process might be more fruitful, giving voice to social robots based on biological rules and dog communicative behaviours (Korcsok et al. 2020, 2024).

## Conclusions for future biology—The dogs bark, but the caravan moves on

One lesson is more for basic research. Despite our expanding knowledge of the evolution and underlying mechanisms of speech-readiness and the growing list of species that exhibit different levels of these capacities, we are still only scratching the surface of how speech might have evolved in humans. For one, this is because we cannot test humans extensively to decipher which selective forces induced the emergence of abilities involved in speech production and perception. Using the available methodological toolbox might be both realistically impossible and unethical (e.g. running experiments that manipulate selective pressures or testing environmental and genetic effects), but we also have no access to a Homo species lacking speech, obviously. Second, it is true that extending the range of search for other species that bear abilities involved in speech has the potential to shed new light on how these abilities emerged through evolution in humans. However, large-scale comparative studies require enormous effort; therefore, it is more plausible to find a few suitable model species. Recently, several novel options, like mice (Fischer and Hammerschmidt 2011), marmosets (Eliades and Miller 2017), or the Bengalese finch (Okanoya 2015), emerged for testing cognitive and vocal capacities presumably involved in speech evolution, but each of these models, although having advantages, also lacks key features paralleling steps of human evolution leading to the appearance of speech. In contrast, dogs, as we saw above, during their evolution, were embedded in human society, and due to similar selective pressures, enhanced and even might have acquired similar abilities that are not just helping them to navigate in the human social environment but also hypothesised to be among the key elements of humans’ speech-readiness. Thus, although dogs certainly will not suddenly acquire speech and language, they provide an excellent opportunity for us to peek into the early stages of speech evolution. Exploring how domestication might have altered dogs, identifying genetic changes that lead to alterations in the vocal repertoire and vocal development, and revealing neural processes and abilities that parallel human capabilities involved in speech processing may all help shed new light on speech evolution. There are indications that dogs can learn to vocalise on command,

The second lesson might be helpful in the applied field of social robotics. One major challenge in this fast-developing area is how we can design robots, particularly their behaviours, to ensure functionality while remaining easily acceptable for humans without any unique expertise (Kubinyi et al. 2010; Faragó et al. 2014b). A social robot should be able to interact with children or seniors smoothly and should not require extensive learning from these users. Dogs undoubtedly excel in this: they understand us very well, and we also understand them surprisingly well, given how far our evolutionary paths diverged. Thus, if we can model the behaviour, communicative, and cognitive abilities of social robots based on dog–human interactions, we can have a chance to get successful artificial companions (Clavel et al. 2013; Wiese et al. 2017; Konok et al. 2018). Although there is no aim to replace dogs with artificial agents (Konok et al. 2018), in some scenarios where service dogs cannot be used (in hospitals, e.g.), such social robots might undoubtedly be advantageous. Thus, we can conclude that instead of redesigning dogs into a novel species by selective breeding for speech, we should equip social robots with abilities and a voice to better integrate them into our lives, based on what we can learn from dogs.
